# Restrict for Health or for Thinness? Liking but Not Approach Bias Differentiates Orthorexic Eating Behavior From Anorexia Nervosa in Responses to High‐Caloric Healthy vs. High‐Caloric Unhealthy Foods

**DOI:** 10.1002/eat.70162

**Published:** 2026-07-07

**Authors:** Hannah van Alebeek, Mareike Röttger, Jana Strahler, Katharina Eichin, Isabella Forwick, Jens Blechert, Ulrich Voderholzer, Julia Reichenberger

**Affiliations:** ^1^ Department of Psychology, Centre for Cognitive Neuroscience Paris Lodron University of Salzburg Salzburg Austria; ^2^ Sportpsychology, Department of Sport and Sport Science University of Freiburg Freiburg Germany; ^3^ Department of Psychology Johannes Kepler University Linz Austria; ^4^ Schoen Clinic Roseneck Prien Germany; ^5^ Department of Psychiatry and Psychotherapy University Hospital, Ludwig Maximilian University Munich Munich Germany; ^6^ Department of Psychology Ludwig Maximilian University Munich Munich Germany

**Keywords:** anorexia nervosa, approach bias, approach‐avoidance task, calories, eating disorders, food, healthiness, liking, orthorexia nervosa, orthorexic

## Abstract

**Objective:**

Individuals with high levels of orthorexic eating behavior (OEB) show a (pathological) fixation on healthy eating. Shared characteristics of OEB with individuals with anorexia nervosa (AN) raised debate over whether orthorexia nervosa constitutes a distinct disorder. Distinctions may lie in preference for and approach behavior toward foods differing in calorie content and healthiness. We expected reduced liking and weaker approach biases in AN and OEB compared with controls; specifically for high‐caloric foods in AN and for unhealthy foods in OEB.

**Method:**

Thirty‐five self‐reported AN, forty non‐AN individuals who scored > 30 on the Düsseldorf Orthorexia Scale (OEB), and 101 individuals with no eating disorder history (controls) completed liking ratings and an Approach‐Avoidance Task with food stimuli varying in calorie content (high vs. low) and, for high‐calorie items, healthiness (healthy vs. unhealthy).

**Results:**

Compared to controls, AN reported lower liking for all high‐caloric foods, while OEB reported lower liking only for high‐caloric, *unhealthy* foods. Accordingly, OEB also liked high‐caloric healthy foods more than AN. AN showed weaker approach bias than controls (mainly driven by high‐caloric unhealthy foods). OEB descriptively lay between both other groups, with no significant differences in approach bias to either.

**Conclusion:**

Explicit liking ratings distinguished AN from OEB aligning with their respective motivation to reduce high‐caloric versus unhealthy food intake. Reduced approach bias was specific to AN, possibly reflecting rapid engagement of approach‐avoidance responses to easy‐to‐categorize high‐caloric unhealthy foods. Contrary, health‐focused processing in OEB may require slower deliberations that are not captured by approach‐avoidance tasks.

## Summary

- There is an ongoing discussion concerning whether orthorexia nervosa, characterized by a (pathological) fixation on healthy eating, represents a distinct phenomenon from anorexia nervosa.
- This study was the first to examine both liking ratings and approach biases toward foods differing in calorie content and healthiness in individuals with anorexia nervosa (AN), high levels of orthorexic eating behavior (OEB), and controls (CG), distinguishing low‐caloric healthy, high‐caloric healthy, and high‐caloric unhealthy foods.
- As expected, AN reported lower liking for high‐caloric foods (healthy and unhealthy) than CG, while OEB reported lower liking only for high‐caloric unhealthy foods. Only AN exhibited reduced approach bias overall, particularly for high‐caloric unhealthy foods; no differences were observed for other food categories or in OEB.
- Clinically, approach bias modification could be a promising intervention for high‐caloric *unhealthy* foods in AN, whereas this approach may be generally less relevant for OEB.

## 1 Introduction

While following a healthy diet is widely recognized as an important lifestyle factor in the prevention of various diseases (Sofi et al. 2013; WHO 2025), an obsessive fixation on healthy eating can lead to functional impairment in other life domains and even to malnutrition (Horovitz and Argyrides 2023). This fixation on the quality and purity of food is referred to as orthorexic eating behavior (individuals with high levels of orthorexic eating behavior = OEB), and as orthorexia nervosa when it reaches a pathological level (Horovitz and Argyrides 2023). Diagnostic criteria for orthorexia nervosa have been proposed, emphasizing its potential clinical significance and the possibility of future classification as a distinct eating disorder (Cena et al. 2019; Donini et al. 2022). However, since orthorexia nervosa is not currently included in diagnostic systems such as the DSM‐5, ICD‐10, or ICD‐11, it is usually assessed with questionnaires (Meule et al. 2020), some of which also include cutoffs for potentially pathological orthorexic eating behavior (e.g., Barthels et al. 2015; Donini et al. 2005).

Some experts question the independence of orthorexia nervosa because of its overlap with other disorders (Koven and Abry 2015). Especially anorexia nervosa (individuals with anorexia nervosa = AN), which is characterized by low body weight, fear of weight gain, a persistent pattern of restrictive eating, and preoccupation with body shape or weight (American Psychiatric Association 2013), is frequently associated with high levels of orthorexic eating behavior (Cosh et al. 2023). In turn, OEB report concerns related to thinness and muscularity‐oriented eating (Wachten and Strahler 2026) and respond to eating disorder‐specific treatment, although such treatment does not specifically target orthorexic eating behavior (Hessler‐Kaufmann et al. 2021). This overlap raises the question of whether OEB experience a distinct disorder or merely a variant of anorexia nervosa (Bhattacharya et al. 2022). From a phenomenological perspective, this overlap becomes particularly evident, as anorexia nervosa and orthorexia nervosa share several psychological and behavioral characteristics, including high trait perfectionism and anxiety, trivializing the severity and associated impairments, the potential for significant weight loss, and feelings of guilt and self‐control failure when eating behavior does not align with self‐imposed restrictions (Koven and Abry 2015; McComb and Mills 2019).

Both OEB and AN also self‐impose rigid food restriction, yet the focus differs according to definitions: OEB primarily restrict foods they consider unhealthy, reflecting a concern with food *quality*. AN restrict high‐caloric or foods in general, reflecting a concern for energy *quantity* (Koven and Abry 2015). Thus, the two phenomena may be distinguished by their responses to foods systematically varying in perceived healthiness versus calorie content.

### 1.1 Food‐Specific Liking Ratings and Biases in OEB and AN

There are several ways to measure responses to foods: One approach is to directly ask participants how much they like, crave or desire specific foods shown in images, while a less explicit possibility is to investigate the behavioral readiness to approach food stimuli, the so‐called approach bias. While food ratings are typically collected without time pressure and thus permit reflection processes on different aspects of the food, approach bias is typically measured via reaction‐time tasks in which participants are instructed to rapidly approach and avoid foods (as well as neutral objects). Responses to these two measurement possibilities overlap, in that approach bias tends to be stronger for specifically those foods that are rated higher in desire to eat or liking (Galler et al. 2022; Kahveci et al. 2020, 2021; van Alebeek et al. 2023).

Thus, one would expect to see potential differences between AN and OEB both when measured through explicit liking ratings as well as when measured through approach bias. However, in cases where liking is at least partly shaped by more complex considerations regarding freshness, nutrient content, degree of processing, or calories, it may be possible that these deliberations are impacting the liking ratings, but are less influential for rapid, time‐pressured responses. This might then result in *less* pronounced differences in approach biases than liking ratings, especially when individuals require time to categorize food stimuli. At the same time, approach biases should, due to the time pressure, be less influenced by potentially distorting processes such as social desirability (which might be relevant due to a higher tendency for perfectionistic self‐promotion within AN and OEB; Casale et al. 2024; Pratt et al. 2022). This might result in *more* pronounced differences in approach bias than liking ratings, especially when individuals are unable or unwilling to report their true preferences. To our knowledge, no studies have directly compared OEB and AN in their reactions to food stimuli. However, there are studies targeting one of these two phenomena.

Compared to controls, AN like low‐caloric foods more than high‐caloric foods (Cowdrey et al. 2013; Lloyd and Steinglass 2018; Paslakis et al. 2016). Evidence for approach bias is mixed, with most studies reporting food avoidance regardless of calorie density (Meregalli et al. 2024; Neimeijer et al. 2019; Paslakis et al. 2016) and one study reporting food avoidance specifically for high‐caloric stimuli (Dimakopoulou et al. 2025). Regarding OEB, one study showed that higher orthorexic eating behavior was associated with higher desire for healthy foods, with no corresponding association for unhealthy foods (Gumussoy et al. 2025). No study has yet investigated approach bias within OEB. Thus, although OEB and AN might be dissociated in their responses to specific food types, no study has directly compared their liking ratings or approach biases. Addressing this question requires an image set that disentangles the naturalistic confounding of perceived healthiness with low calorie density. Because popular ‘healthy’ foods (e.g., fruits, vegetable) tend to be lower in calories than popular “unhealthy” foods (e.g., pizza, burger, fries), careful stimulus selection is essential to isolate calorie‐specific versus health‐specific response patterns that might provide more insight into differences and similarities in AN and OEB.

### 1.2 The Current Study

The current study directly compared liking ratings and approach biases of OEB and AN against a control group (CG). The food stimuli used in the study varied both on calorie content (high vs. low) and, within the high‐calorie category, on perceived healthiness (unhealthy vs. healthy). We hypothesized that AN and OEB would show lower liking ratings and weaker approach biases toward foods overall compared with CG. For AN, these group differences to CG should be mainly driven by high‐caloric foods (healthy and unhealthy). For OEB, these group differences should be mainly driven by unhealthy foods regardless of calorie density.

## 2 Method

### 2.1 Recruitment of Participants

OEB and CG were recruited from German and Austrian universities as well as in gyms and organic supermarkets, where an above‐average number of OEB was expected (Hafstad et al. 2023; Voglino et al. 2021). The study was also advertised on six Instagram accounts revolving around eating disorder recovery and healthy eating (Supporting Information: Appendix A). Additionally, OEB were recruited through the online recruitment service (Probando GmbH, n.d.). AN were recruited from the waiting list of an eating disorder clinic, the Schönklinik Roseneck. Inclusion criteria were a minimum age of 18 years and fluent German language skills. The AN group additionally required a current diagnosis of anorexia nervosa. Although no diagnostic interview was conducted, diagnostic validity is likely, as participants reported a prior clinical diagnosis and were recruited from a treatment waitlist requiring diagnostic confirmation. The OEB group required a score above the cut‐off of 30 on the Düsseldorf Orthorexia Scale (DOS; Barthels et al. 2015) but no self‐reported diagnosis of anorexia nervosa. Since the DOS assesses aspects of OEB also inherent to AN (e.g., “I have set up dietary rules”), it was not feasible to exclude participants with high DOS scores from AN without substantially reducing this group's sample size. For the CG, inclusion required reporting no current or past eating disorder diagnosis and a DOS score below 30. To examine the robustness of our results under stricter, though potentially less externally valid, group assignment criteria, we conducted a sensitivity analysis (Supporting Information: Appendix B): Individuals at risk for orthorexia nervosa (i.e., DOS ≥ 25 instead of ≥ 30) were additionally excluded from the CG, and participants with a past eating disorder diagnosis were excluded from OEB (12.5% of the original OEB had a past anorexia nervosa diagnosis). The results from this sensitivity analysis closely mirrored the main findings reported below. No a priori power analysis was conducted.

### 2.2 Procedure

The study received ethical approval from the ethics board of the Justus Liebig University Giessen (ref number: 2020‐0049), and data were collected between 2021 and 2024. After providing informed consent, participants completed an online screening via SosciSurvey (Leiner 2024). This screening assessed sociodemographic data and included the DOS. Also, current (i.e., within the last 3 months) and past (i.e., more than 3 months ago) eating disorder diagnoses were assessed using the response options “Yes, anorexia nervosa,” “Yes, bulimia nervosa,” “Yes, binge eating disorder,” “Yes, unspecified eating disorder,” and “No.”

The study was part of a larger project. Following screening, participants received separate links for two experimental sessions conducted via the online platform Gorilla (Anwyl‐Irvine et al. 2020, 2021). Sessions were conducted 1 day apart and within one to 2 h after a meal for standardization purposes (since approach bias fluctuates with hunger; Zech et al. 2023). The approach‐avoidance task (AAT) and food image ratings were performed during the second experimental session. The sessions were linked based on a self‐generated participant code. After completing all study components, participants were remunerated with 30 euros. The first experimental session as well as additional questionnaires and experiments of the three study parts are described in Supporting Information: Appendix A.

### 2.3 Düsseldorf Orthorexia Scale (DOS)

Orthorexic eating behavior was assessed with the DOS containing 10 items (e.g., “I can only enjoy food if it is healthy”). Items were rated on 4‐point Likert scales from 1 (does not apply to me) to 4 (applies to me), yielding total sum scores between 10 and 40 with excellent internal consistency in the current sample (Cronbach's *α* = 0.94). Following Barthels et al. (2015), a score ≥ 30 was used as the cut‐off for OEB.

### 2.4 Stimuli Selection and Ratings

Fifteen of the 18 food images used in the AAT were selected from the *food.pics_extended database* (Blechert et al. 2019), while three high‐caloric healthy foods were selected from online resources. The foods were selected to differ in their calorie content and perceived healthiness, yielding three food categories of six images each (all food stimuli are listed in Supporting Information: Appendix C): (1) low‐caloric healthy foods (e.g., salad; *M* = 27.83 kcal, SD = 11.65), (2) high‐caloric unhealthy foods (e.g., milk chocolate; *M* = 305.00 kcal, SD = 167.01), and (3) high‐caloric healthy foods (e.g., nuts; *M* = 325.17 kcal, SD = 185.62). Control stimuli in the AAT included 18 office objects such as ruler, pencil, or stapler selected from the *food.pics_extended database* (Blechert et al. 2019) and the *FRIDa image database* (Foroni et al. 2013).

As the healthiness selection was based on our subjective perceptions of culturally shared notions of “healthy” versus “unhealthy” foods, we validated if participants perceived the three food categories as intended: each food image was rated regarding its perceived calorie content (“How do you estimate the calorie content of this food (per 100g)?” from 0 (“no calories at all”) to 100 (“a lot of calories”)) and healthiness (“How healthy do you think this food is?” from 0 (“not healthy at all”) to 100 (“very healthy”)). As intended (see Figure 1), high‐caloric unhealthy foods were rated as highest in calories and lowest in healthiness, low‐caloric healthy foods as lowest in calories and highest in healthiness, and high‐caloric healthy foods as intermediate on both dimensions. Supporting Information: Appendix D presents ratings separately for each diagnostic group.

**FIGURE 1:**
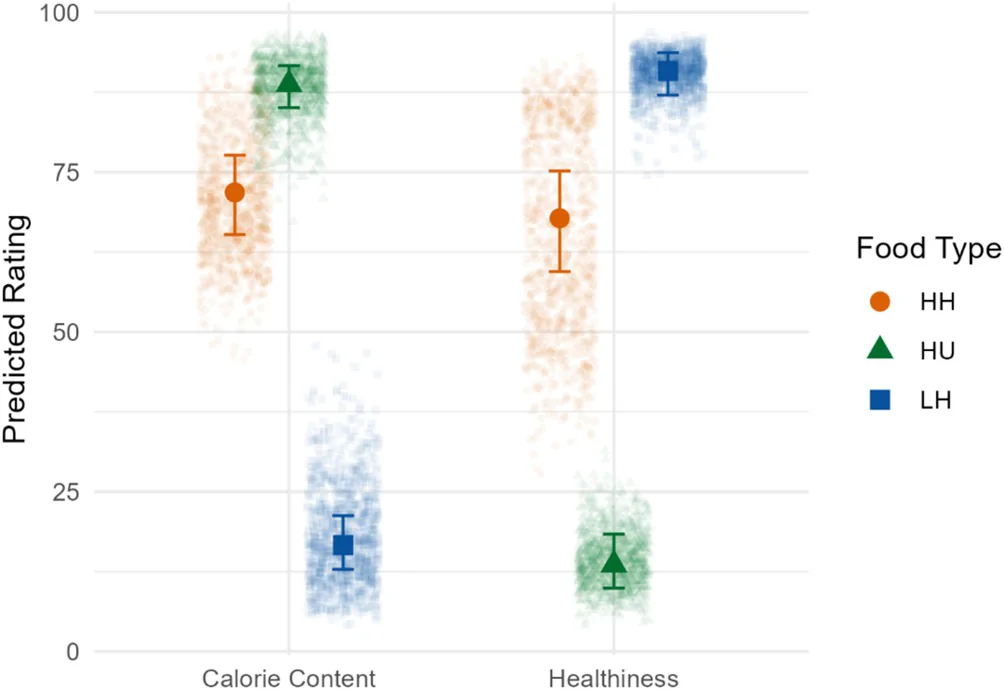
Ratings of calorie content and healthiness by stimulus category in the current sample. The error bars represent confidence intervals. HH = high‐caloric healthy foods; HU = high‐caloric unhealthy foods; LH = low‐caloric healthy foods.

In addition, as one of the primary outcome measures, participants rated each food image on liking (“How much do you like this food?”) with answer options ranging from 0 (“not at all”) to 100 (“very much”).

### 2.5 Approach‐Avoidance Task (AAT)

Food approach biases were measured using a modified version of the manikin task, originally developed by de Houwer et al. (2001), illustrated in Figure 2. We employed feature‐relevant task instructions (motivated by Kahveci et al. 2023; Lender et al. 2018; Phaf et al. 2014), requiring participants to approach or avoid stimuli based on their category (i.e., foods vs. objects) rather than irrelevant perceptual features (e.g., portrait vs. landscape orientation). Thereby, the distinction between foods and objects was task‐relevant, but the distinction between the three food categories was task‐irrelevant.

**FIGURE 2:**
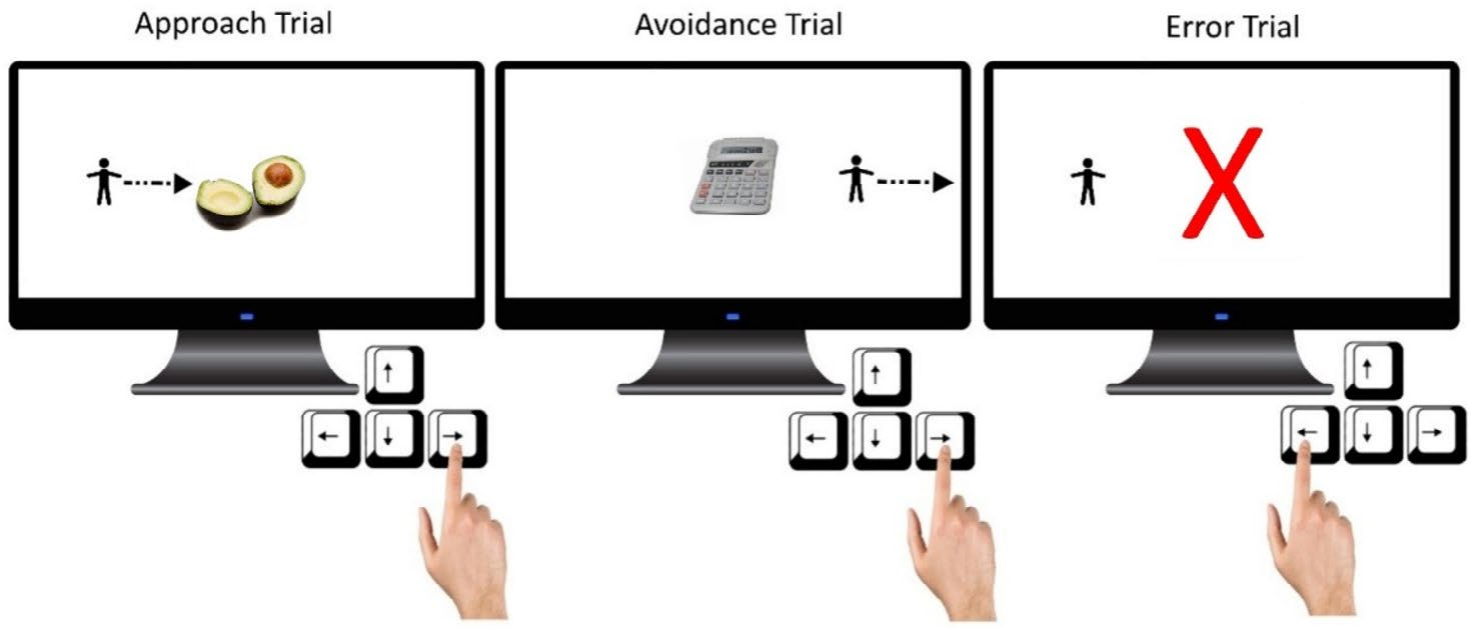
Illustration of the approach–avoidance task for a high‐caloric healthy food under bias‐congruent instructions. Figure adapted from van Alebeek et al. (2025). In each trial, a picture appeared in the centre of the screen, accompanied by a manikin displayed either to its left or right, with the position randomized with equal probability. Participants used the keyboard arrow keys to either approach the picture (by moving the manikin toward it) or avoid it (by moving the manikin away toward the screen border). Once the manikin reached the picture or screen boundary, the stimulus disappeared, and the next trial began. Errors were indicated by a red “X” displayed for one second. Key presses can sufficiently capture approach biases, as they emerge before motor execution and primarily depend on how the movement is interpreted (approach vs. avoidance), irrespective of the specific muscles used (Kahveci et al. 2021; Rinck and Becker 2007).

The task began with 16 practice trials depicting images of dogs and butterflies. Participants then completed eight experimental blocks of 36 trials each, for a total of 288 trials. Each block contained all 18 food and 18 control images once, presented in randomized order. Task instructions alternated across blocks, such that participants were required either to approach food and avoid objects (i.e., bias‐congruent instructions) or to do the reverse (i.e., bias‐incongruent instructions) to minimize potential block‐order effects (usually due to overall speeding or slowing; van Alebeek et al. 2021). Block order was counterbalanced: half of the participants approached foods first, while the other half approached control images first.

We calculated Spearman–Brown corrected bootstrapped split‐half reliabilities for bias scores based on 1000 random splits (Kahveci 2022). Reliability was acceptable and similar to that of previous online and lab‐based AATs (van Alebeek et al. 2023, 2025) for biases toward all food types combined (*r*_SB_ = 0.73), and somewhat lower for specific categories (probably due to lower trial number; Hedge et al. 2018): low‐caloric healthy foods (*r*_SB_ = 0.57), high‐caloric unhealthy foods (*r*_SB_ = 0.58), and high‐caloric healthy foods (*r*_SB_ = 0.56).

### 2.6 Statistical Analysis

The data and analysis scripts are available online (https://osf.io/f8rb4/). Analyses for approach bias and liking ratings were first conducted across all food stimuli and then repeated in separate models for each food category. For the separate models, the significance threshold was Bonferroni‐corrected from α = 5% to α = 1.67%. One BMI value (60.1 kg/m^2^) was considered implausible and likely reflecting a reporting error in the online sample, and was thus set to missing.

To test whether approach bias differed between CG and AN and between CG and OEB, trial‐level AAT RTs were predicted using the movement × stimulus type × group interaction. Categorical predictors were dummy coded: movement type (0 = avoidance, 1 = approach), stimulus type (0 = object, 1 = food), and participant group, with the CG serving as the reference category (Dummy 1: 0 = CG, 1 = AN; Dummy 2: 0 = CG, 1 = OEB). RT data was preprocessed as recommended by Kahveci et al. (2023): practice trials were omitted, and task trials were excluded if errors occurred, and if RTs were under 200 ms or over 2000 ms. Any remaining trials deviating more than three standard deviations from the individual mean RT were also excluded as well as participants with over 35% excluded trials. RTs are expressed in hertz (Hz), such that higher values indicate more responses per second (i.e., faster responses). Since AAT trials were nested within participants and food images, we estimated multilevel models including random effects for participants and stimuli. Because the maximal random‐effects structure did not converge, we adopted the most complex structure that allowed convergence by excluding the random slope for movement by stimulus:

(1) $$\begin{array}{c} \mathrm{inv}.\mathrm{RTs}\sim \text{Movement}\;\left(\text{avoid}\;\mathrm{vs}.\;\text{approach}\right)\\ \times \text{Stimulus}\;\left(\text{object}\;\mathrm{vs}.\;\text{food}\right)\times \text{Dummy}\;1\;\left(\mathrm{CG}\;\mathrm{vs}.\;\text{AN}\right)\\ +\text{Movement}\;\left(\text{avoid}\;\mathrm{vs}.\;\text{approach}\right)\times \text{Stimulus}\;\left(\text{object}\;\mathrm{vs}.\;\text{food}\right)\\ \times \text{Dummy}\;2\;\left(\mathrm{CG}\;\mathrm{vs}.\mathrm{OEB}\right)\\ +\left(\text{Movement}\times \text{Stimulus}\times \text{Group}\;|\;\text{Subject}\right)\\ +\left(1\;|\;\text{Stimulus}\right) \end{array}$$

To examine whether food liking differed between CG, AN, and OEB, we predicted image‐level liking ratings using participant group as the predictor (same dummy coding). Because liking ratings were clustered at the extremes (i.e., participants mostly either liked or disliked specific foods on the scale from 0 to 100), we rescaled them to a 0–1 range and modeled them using a beta distribution. To account for the nested data structure, we included random intercepts for both participants and stimuli:

(2) $$\begin{array}{c} \text{LikingRatings}\sim \text{Dummy}\;1\;\left(\mathrm{CG}\;\mathrm{vs}.\;\text{AN}\right)\\ +\text{Dummy}\;2\;\left(\mathrm{CG}\;\mathrm{vs}.\mathrm{OEB}\right)\\ +\left(1\;|\;\text{Subject}\right)+\left(1\;|\;\text{Stimulus}\right) \end{array}$$

## 3 Results

### 3.1 Sample Characteristics

Of the 253 participants starting the second experimental session, 240 completed the AAT, and 205 of these could be uniquely matched to the initial screening used for group assignment. Seven participants were excluded due to 35% excluded trials in the AAT and one was excluded for being younger than 18 years (predefined exclusion criteria). Among the remaining sample, 35 could be classified as AN, 39 as OEB and 101 as CG. The 22 participants reporting a current or past diagnosis of an eating disorder not relevant to the present study (e.g., bulimia nervosa or binge eating disorder) and having a DOS score below 30 were excluded from further analyses. Group differences in age, BMI, and DOS scores are reported in Table 1.

**TABLE 1:** Comparison of the sample's characteristics.

Variable	Groups				*X* (df)	*p*
	Overall *N* = 175	CG *N* = 101	AN *N* = 35	OEB *N* = 39		
	*M* (SD) [min − max]	*M* (SD) [min − max]	*M* (SD) [min − max]	*M* (SD) [min − max]		
Women (%)	86.86%	86.14%^a^	97.14%^a^	80.00%^a^	4.96 (2)	0.076
Age	25.71 (8.81) [18–61]	24.68 (7.92)^a^ [18–61]	23.60 (6.8)^a^ [18–39]	30.28 (11)^b^ [15–61]	16.58 (2)	< 0.001
BMI	21.23 (4.19) [13.03–36.03]	22.27 (3.89)^a^ [15.78–36.03]	16.65 (2.78)^b^ [13.03–25.97]	21.92 (3.61)^a^ [15.54–34.48]	47.85 (2)	< 0.001
DOS	24 (8.52) [10–39]	17.75 (4.48)^b^ [10–29]	31.77 (5.49)^a^ [17–39]	33.21 (2.48)^a^ [30–38]	118.64 (2)	< 0.001

*Note:* Group differences were observed with Bonferroni‐adjusted post hoc Kruskal–Wallis rank‐sum tests due to violations of homogeneity of variance for age, BMI, and DOS scores. Means with different superscripts differ significantly at *p* < 0.05: OEB were older than both AN and CG, AN had a lower BMI than OEB and CG, and DOS scores were lower in CG than in AN and OEB (all *p*_adj_ < 0.01). Given unexpected age differences and some overlap in BMI ranges, sensitivity analyses controlling for age and applying either the WHO cut‐off for underweight (i.e., BMI < 18.5) or the DSM‐5 cut‐off for moderate AN (i.e., BMI < 17) were conducted and yielded results consistent with those reported in the main analyses (see Supporting Information: Appendix B).

Abbreviations: AN = individuals with anorexia nervosa; BMI = body mass index; CG = control group; DOS = Düsseldorf Orthorexia scale; OEB = individuals with high levels of orthorexic eating behavior.

### 3.2 Main Analysis

#### 3.2.1 Explicit Liking Ratings

Compared with CG, AN rated food stimuli significantly lower, whereas OEB did not (see Table 2). When using separate models for each food category, differences between AN and OEB emerged specifically for high‐caloric healthy foods (see Figure 3). Both AN and OEB reported lower liking for *high‐caloric unhealthy* foods relative to CG; however, only AN reported lower liking for *high‐caloric healthy* foods compared with OEB and CG. OEB also reported higher liking for *low‐caloric healthy* foods compared with CG, but this difference was not significant at the corrected alpha level. AN and OEB did not differ in their liking for high‐caloric unhealthy and low‐caloric healthy foods. Exploratory multilevel correlations on the image level additionally showed that the link between liking and healthiness ratings was strongest in OEB (see Supporting Information: Appendix E).

**TABLE 2:** Differences between groups in explicit liking ratings.

Food category	AN vs. CG					OEB vs. CG					AN vs. OEB				
	*b*	SE	*z*	*p*	*d*	*b*	SE	*z*	*p*	*d*	*b*	SE	*z*	*p*	*d*
All food stimuli	−0.36	0.10	−3.79	**< 0.001**	−0.35	−0.08	0.09	−0.87	0.384	−0.13	−0.16	0.11	−1.51	0.132	−0.21
High‐caloric unhealthy	−0.69	0.16	−4.42	**< 0.001**	−0.58	−0.57	0.15	−3.75	**< 0.001**	−0.47	−0.13	0.21	−0.61	0.544	−0.11
High‐caloric healthy	−0.55	0.14	−3.86	**< 0.001**	−0.49	0	0.14	−0.02	0.982	−0.07	−0.55	0.20	−2.80	**0.** **005**	−0.41
Low‐caloric healthy	0.10	0.13	0.72	0.474	0.03	0.29	0.13	2.26	0.024	0.22	−0.19	0.14	−1.31	0.189	−0.19

*Note:* *d* = Cohen's *d*. Bold values indicate significance at a Bonferroni‐corrected alpha‐level of 1.67%.

Abbreviations: AN = individuals with anorexia nervosa; CG = control group; OEB = individuals with high levels of orthorexic eating behavior.

**FIGURE 3:**
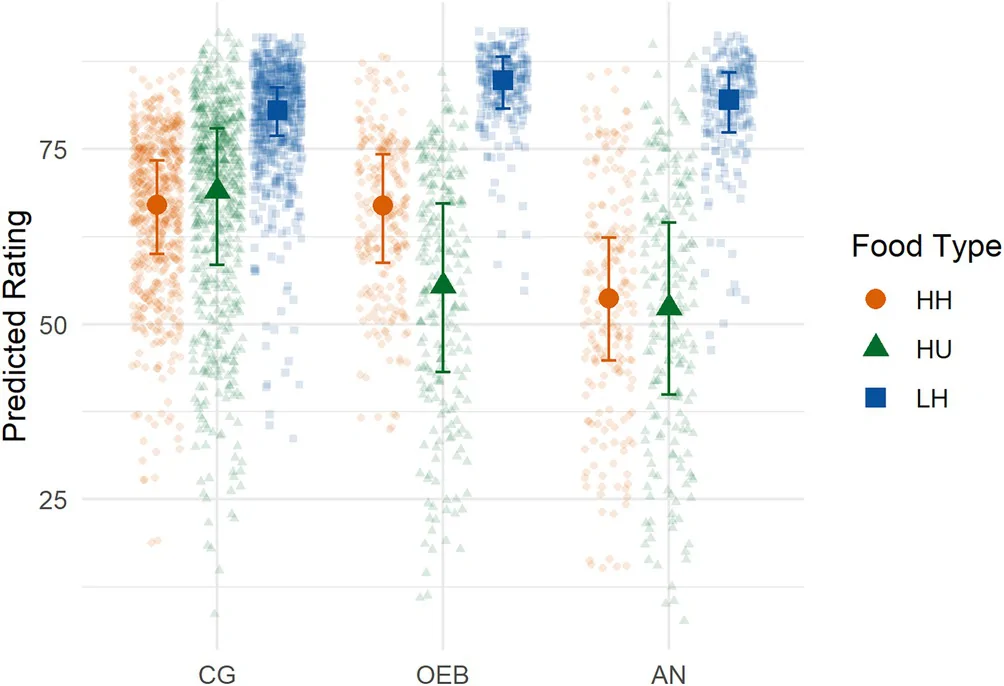
Predicted liking ratings by participant group. The error bars represent confidence intervals. AN = individuals with anorexia nervosa; CG = control group; HH = high‐caloric healthy foods; HU = high‐caloric unhealthy foods; LH = low‐caloric healthy foods; OEB = individuals with high levels of orthorexic eating behavior.

#### 3.2.2 Approach Bias

We examined whether AN or OEB differ in their approach bias to foods in general from CG using 3‐way interactions (Stimulus × Movement × Group). We found a significant 3‐way interaction between the approach bias (Stimulus × Movement) and the presence of AN (*b* = −0.06, *β* = −0.15, SE = 0.03, *t*(169.89) = −2.08, *p* = 0.039), but no significant 3‐way interaction between the approach bias and the presence of OEB (*b* = −0.02, *β* = −0.06, SE = 0.03, *t*(170.08) = −0.82, *p* = 0.415). Accordingly, approach bias differed between CG and AN but not between CG and OEB. As shown in Figure 4, CGs approached foods faster than objects and avoided foods more slowly, whereas this approach bias was significantly attenuated in AN.

**FIGURE 4:**
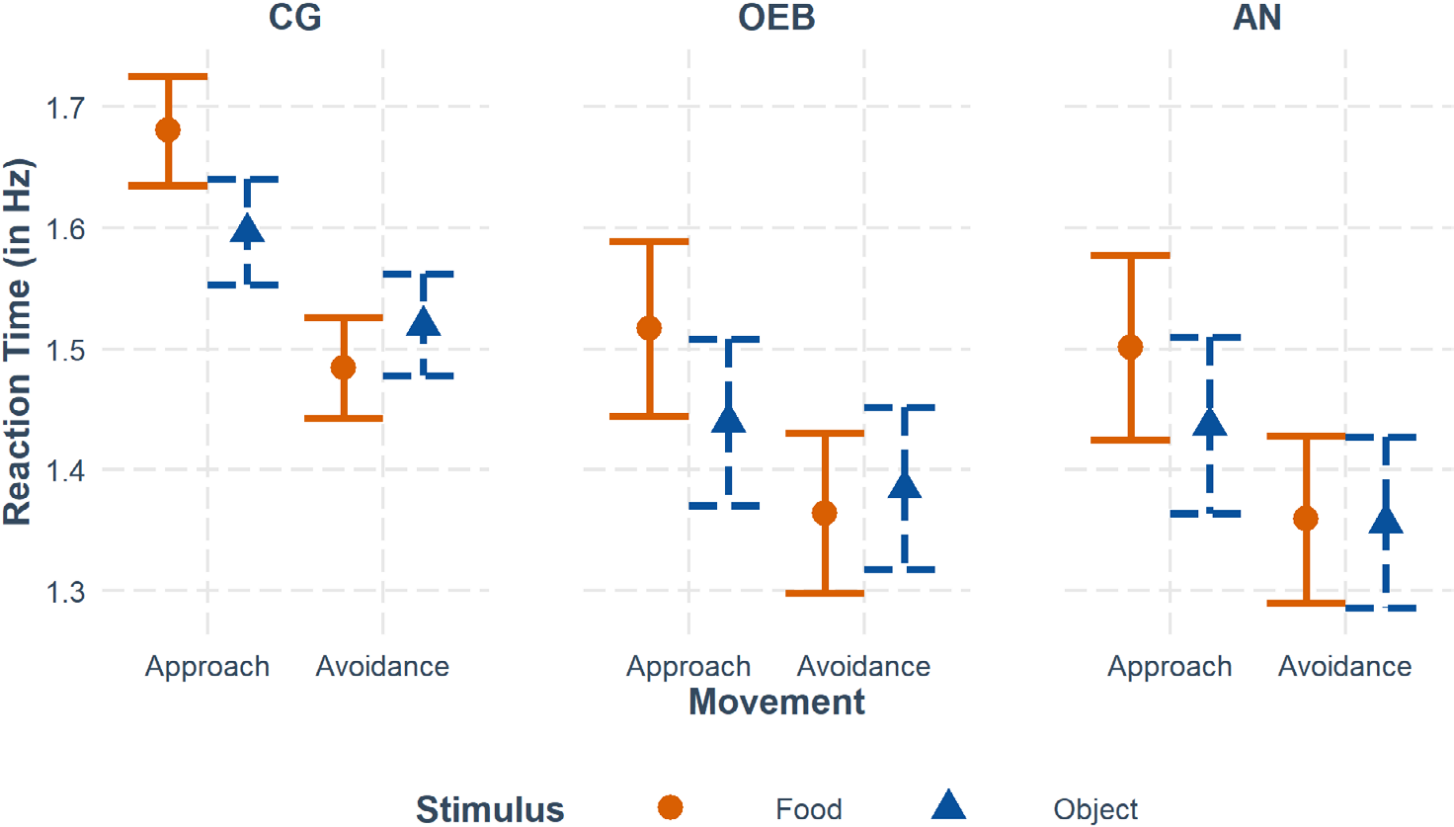
Reduced approach bias for foods in individuals with anorexia nervosa (AN) relative to the control group (CG). The error bars represent confidence intervals. Higher values in the reaction time variable indicate more responses per second (i.e., faster responses). The approach bias is calculated with the interaction of stimulus type (i.e., food vs. object) and movement direction (i.e., approach vs. avoidance). Approach bias in AN is reduced compared to CG, because in AN the difference between approach and avoidance reaction times for foods is closer to the difference between approach and avoidance reaction times in objects. AN = individuals with anorexia nervosa; CG = control group; OEB = individuals with high levels of orthorexic eating behavior.

We further investigated group differences in approach bias to high‐caloric healthy, high‐caloric unhealthy, and low‐caloric healthy foods using three separate models. The 3‐way interaction for AN vs. CG was significant for high‐caloric unhealthy but not for high‐ or low‐caloric healthy foods (see Table 3). Specifically, AN had weaker approach biases to high‐caloric unhealthy foods than CG, whereas no group differences were observed for the other two food categories. For OEB vs. CG, there was, like for the overall food bias, no significant difference in approach bias for the different food types.

**TABLE 3:** Interaction between approach bias (movement × stimulus) and participant group per food type.

Predictors	High‐caloric healthy foods				High‐caloric unhealthy foods				Low‐caloric healthy foods			
	*b*	*β*	*t*	*p*	*b*	*β*	*t*	*p*	*b*	*β*	*t*	*p*
Movement	0.08	0.20	8.00	**< 0.001**	0.08	0.20	8.01	**< 0.001**	0.08	0.20	8.02	**< 0.001**
Stimulus	−0.05	−0.12	−3.73	**< 0.001**	−0.04	−0.10	−2.67	**0.008**	−0.02	−0.06	−1.72	0.086
OEB	−0.14	−0.36	−3.40	**0.001**	−0.14	−0.36	−3.43	**0.001**	−0.14	−0.36	−3.41	**0.001**
AN	−0.16	−0.43	−3.96	**< 0.001**	−0.16	−0.43	−3.96	**< 0.001**	−0.16	−0.43	−3.96	**< 0.001**
Movement × Stimulus	0.12	0.31	7.54	**< 0.001**	0.11	0.29	6.99	**< 0.001**	0.13	0.34	8.08	**< 0.001**
Movement × OEB	−0.02	−0.06	−1.20	0.232	−0.02	−0.06	−1.16	0.246	−0.02	−0.06	−1.19	0.234
Movement × AN	0	0.01	0.19	0.850	0	0.01	0.18	0.859	0	0.01	0.19	0.847
Stimulus × OEB	0.03	0.07	1.56	0.120	0.02	0.05	1.07	0.285	0	0	−0.07	0.944
Stimulus × AN	0.03	0.08	1.77	0.076	0.05	0.14	2.86	**0.004**	0.03	0.09	1.84	0.066
Movement × Stimulus × OEB	−0.03	−0.07	−0.90	0.368	−0.02	−0.07	−0.83	0.408	−0.01	−0.03	−0.32	0.750
Movement × Stimulus × AN	−0.03	−0.08	−1.01	0.314	−0.08	−0.21	−2.52	**0.012**	−0.06	−0.16	−1.96	0.050
*N*	175_subject_				175_subject_				175_subject_			
	24_Stimulus_				24_Stimulus_				24_Stimulus_			
Observations	30,337				30,314				30,307			

*Note:* Bold values indicate significance at a Bonferroni‐corrected alpha‐level of 1.67%. AN = dummy variable for control group vs. individuals with anorexia nervosa, OEB = dummy variable control group vs. individuals with high levels of orthorexic eating behavior. *β* = standardized coefficient.

When specifically testing approach bias differences between AN and OEB, there was neither a significant 3‐way interaction for the model on all foods combined (*b* = −0.04, *β* = −0.05, SE = 0.03, *t*(72.19) = −1.09, *p* = 0.281), nor for the models focusing on high‐caloric healthy (*b* = 0, *β* = −0.01, SE = 0.04, *t*(71.64) = −0.11, *p* = 0.912), high‐caloric unhealthy (*b* = −0.05, *β* = −0.07, SE = 0.04, *t*(72.31) = −1.37, *p* = 0.176), and low‐caloric healthy foods (*b* = −0.05, *β* = −0.07, SE = 0.04, *t*(72.53) = −1.40, *p* = 0.165).

## 4 Discussion

The current study extends previous research by comparing liking ratings and approach bias between AN, OEB, and CG while differentiating between healthiness and calorie content. Our results confirmed expected dissociations between AN and OEB (relative to each other and to CG) for liking ratings but not for approach bias: Liking ratings diverged between the two groups specifically for high‐caloric healthy foods, but approach bias did not diverge specifically for this food category.

### 4.1 Expected Differences in Liking Ratings

In line with the (proposed) diagnostic criteria of AN and OEB, our results suggest that food liking in AN is primarily influenced by calorie content (independent of healthiness) with medium‐sized differences between AN and CG for high‐caloric foods, whereas in OEB it is more strongly shaped by perceived healthiness with small‐ to medium‐sized differences between OEB and CG for high‐caloric unhealthy and low‐caloric healthy foods (Cohen 2013). These results align with our hypothesis and with previous studies showing lower liking and wanting of high‐ versus low‐caloric foods in AN compared with controls (review: Lloyd and Steinglass 2018) and greater desire for healthy but not unhealthy foods in OEB (Gumussoy et al. 2025). Crucially, by disentangling calorie content from perceived healthiness, the present study is the first to demonstrate that these dimensions dissociate AN and OEB directly: group differences emerged for high‐caloric healthy foods but not for high‐caloric unhealthy foods, a distinction that could not be made in previous studies as healthiness and calorie density are usually partially confounded (Godier et al. 2016; Gumussoy et al. 2025).

### 4.2 Smaller Approach Bias for High‐Caloric Unhealthy Foods in AN

We found no alterations in approach bias in OEB relative to CG, neither when we combined the food categories nor when we analyzed them separately. To our knowledge, this is the first study to investigate approach bias within OEB. For AN, we found a lower approach bias for foods in general compared with CG, consistent with previous studies (Meregalli et al. 2024; Neimeijer et al. 2019; Paslakis et al. 2016). When examining food categories separately, approach bias was weaker only for high‐caloric unhealthy foods with a small effect size (Cohen 2013; please note that due to large variances innate to reaction times, interpretations based on conventional effect size guidelines are not applicable), but not for high‐caloric healthy or low‐caloric foods. This pattern suggests that expected (but previously not found) differences between high‐ and low‐calorie foods in AN may emerge when focusing specifically on high‐caloric unhealthy foods.

Interestingly, this pattern of results for separate food categories in AN aligns with our expectation for OEB. An explanation for why these results emerged in AN but not in OEB might lay in the easiness of categorization based on the dimensions of healthiness and calorie content: OEB are less certain when categorizing foods based on healthiness (Lakritz et al. 2025). During reaction time tasks, the time constraint for the deliberate processes involved in this categorization might blur any potential difference between OEB and controls (Lakritz et al. 2022). AN, in contrast, are faster than controls in categorizing foods based on calorie content (Dondzilo et al. 2024). Since calorie content and healthiness are usually confounded, our study might suggest that, when confronted with the time constraints of a reaction‐time based measure, AN tend to use heuristics where they are especially fast in categorizing unhealthy high‐caloric foods as “high‐caloric” (a heuristic also found in the general population; Masterson et al. 2020), while this heuristic cannot be applied to high‐caloric healthy foods, where we see the expected results in the liking ratings only.

### 4.3 Limitations and Strengths

A strength of the current study is the food stimulus selection not only based on calorie content but also based on healthiness. While the healthiness selection was based on the researchers' subjective judgment and not validated beforehand, it was validated by participants' ratings during the study. Since it is unclear which of the many dimensions that encompass “healthiness” (e.g., fiber content, types of fatty acids, degree of processing, or specific vitamins) participants prioritize when evaluating foods, this approach may provide a more ecologically valid stimulus set than relying solely on objective nutritional criteria.

A limitation of the study was that we did not include low‐caloric, unhealthy foods, and thus could not fully cross healthiness with calorie content for the included food categories. This reflected a methodological trade‐off: we aimed to include at least six stimuli per category to ensure that the effects generalize beyond specific items (e.g., biases because specific stimuli such as ice pops are perceived positive independent of their calorie content and healthiness). However, finding enough visually distinguishable and easy‐to‐categorize low‐caloric unhealthy foods was challenging (as classification for e.g., low fat alternatives often depends on packaging or additional nutritional information), leading us to focus on more clearly and visually distinguishable food categories. We encourage further work to identify food stimuli that are generally perceived to fit in this category and incorporate corresponding images into existing food databases.

Another limitation is that the “liking” item captures general food preference but not how much participants are actually willing to eat a given food. As these constructs may diverge, future research should assess both to better disentangle hedonic preference from intake‐related processes. In addition, since we did not conduct a power analysis, it may be possible that non‐significant differences between AN and OEB as well as OEB and CG in approach biases may be a result of non‐sufficient power. Lastly, reliance on self‐reported diagnosis in the AN group may have introduced some diagnostic imprecision (e.g., due to low‐insight). Hence, future research might benefit from using standardized diagnostic interviews and validating groups against additional eating disorder questionnaires.

### 4.4 Implications

The current results have implications for the ongoing debate around a potential differentiation of OEB and AN (Bhattacharya et al. 2022; Gramaglia et al. 2017), as well as further research and treatment trajectories.

Consistent with conceptualizations of AN and OEB as overlapping constructs (Koven and Abry 2015), and with criticism that the DOS does not clearly differentiate between both (Ng et al. 2024), DOS scores were comparable high in the AN and OEB group. This reflects the sampling asymmetry in the current study: We could obtain an OEB group without AN, but not an AN group without OEB. This pattern aligns with the idea that traits of OEB may occur independently at subclinical levels, whereas in AN, these tendencies (potentially in more rigid forms) are almost always present alongside symptoms of anorexia nervosa. Longitudinal designs are needed to clarify whether orthorexia represents a prodromal or residual form of AN in certain cases. The latter might explain our finding that 12.5% of OEB reported a past diagnosis of anorexia nervosa, and evidence of a shift from AN to orthorexia nervosa during recovery (Segura‐Garcia et al. 2015).

Besides the overlap in DOS scores and diagnostic history, we found significant difference between AN and OEB in weight and in liking ratings when considering the food type. Further, even though approach bias did not differ between AN and OEB, the reduced bias in AN compared to CG can be interpreted as unique to AN, as both contrasts were analyzed in the same model controlling for shared variance. Taken together, the current study highlights that, besides diagnostic overlap, AN and OEB show distinct food‐related responses, especially when differentiating foods by calorie content *and* healthiness.

Regarding treatment implications, our result that approach bias differed between AN and CG specifically for high‐caloric *unhealthy* foods might suggest lower treatment ambivalence and refusal in AN (Guarda 2008) when focusing on high‐caloric *healthy* foods during the weight restoration treatment phase. In addition, approach bias modification aimed at increasing engagement with high‐caloric unhealthy foods (Blomberg et al. 2025) could be beneficial in AN, whereas such interventions are not supported for OEB based on the current results.

## Author Contributions

**Isabella Forwick:** investigation, writing – review and editing, project administration. **Julia Reichenberger:** funding acquisition, conceptualization, methodology, investigation, supervision, writing – review and editing, project administration. **Mareike Röttger:** writing – original draft, writing – review and editing. **Ulrich Voderholzer:** writing – review and editing. **Katharina Eichin:** conceptualization, writing – review and editing, methodology. **Hannah van Alebeek:** conceptualization, methodology, software, data curation, supervision, formal analysis, visualization, writing – original draft, writing – review and editing. **Jana Strahler:** funding acquisition, conceptualization, methodology, writing – review and editing. **Jens Blechert:** conceptualization, methodology, supervision, writing – review and editing.

## Lived Experience Involvement Statement

No specific efforts were undertaken to involve persons with lived experience in the study design or execution, or in the preparation of this manuscript.

## Funding

This research was funded by the Peer‐Mentoring Program of the German Society for Behavioral Medicine and Behavior Modification (DGVM). Mareike Röttger is a recipient of a DOC Fellowship of the Austrian Academy of Sciences (ÖAW) at the University of Salzburg. For open access purposes, the author has applied a CC BY public copyright license to any author accepted manuscript version arising from this submission.

## Disclosure

No AI tools were used in the manuscript preparation process.

## Conflicts of Interest

The authors declare no conflicts of interest.

## Supporting information


**Data S1:** eat70162‐sup‐0001‐Supinfo.docx.

## Data Availability

Data, analysis scripts, and the online supplement can be found in the OSF.io repository: https://osf.io/f8rb4/. Versions of the manuscript are published as a pre‐print: https://doi.org/10.31234/osf.io/c5v4e_v1/https://doi.org/10.31234/osf.io/c5v4e_v2.
