# Supplementary material for: Restrict for Health or for Thinness? Liking but Not Approach Bias Differentiates Orthorexic Eating Behavior From Anorexia Nervosa in Responses to High‐Caloric Healthy vs. High‐Caloric Unhealthy Foods
Source: Int J Eat Disord. 2026 Jul 7;59(11):2451–61. doi: 10.1002/eat.70162 (PMC13726449; doi:10.1002/eat.70162)
Supplement: Supplementary file 1 [file EAT-59-2451-s001.docx]

Online Supplement for “Restrict for Health or for Thinness? Liking but Not Approach Bias Differentiates Orthorexic Eating Behavior from Anorexia Nervosa in Responses to High-Caloric Healthy vs. High-Caloric Unhealthy Foods”

Hannah van Alebeek^a*^, Mareike Röttger^a*^, Jana Strahler^b^, Katharina Eichin^c^, Isabella Forwick, Jens Blechert^a^, Ulrich Voderholzer ^d, e^ & Julia Reichenberger^a, f^

a. Department of Psychology, Centre for Cognitive Neuroscience, Paris Lodron University of Salzburg, Salzburg, Austria
b. Sportpsychology, Department of Sport and Sport Science, University of Freiburg, Germany
c. Department of Psychology, Johannes Kepler University, Linz, Austria
d. Schoen Clinic Roseneck, Prien, Germany
e. Department of Psychiatry and Psychotherapy, University Hospital, Ludwig Maximilian University Munich, Germany
f. Department of Psychology, Ludwig Maximilian University Munich, Germany

*Hannah van Alebeek and Mareike Röttger contributed equally and share the first authorship.

Declarations of interest: none

**Appendix A**

**Description of the Procedure of additional Study Parts not relevant for the current Analysis**

The larger project aimed to investigate individuals with high levels of orthorexic eating behavior (OEB), with three overarching goals: 1) identifying neuropsychological correlates of orthorexic eating behavior, including biases, food cue processing, and emotional eating; 2) examining patterns of emotional over- or under-eating in OEB; and 3) differentiating OEB from both individuals with non-pathological interest in healthy eating and individuals with related clinical disorders, particularly anorexia nervosa. To address these aims, the study had the following procedure for finding collaborating Instagram accounts and assessed the following variables in addition to those described in the main manuscript.

To find Instagram accounts that would potentially collaborate for recruitment by sharing the current study, accounts were searched using the terms “healthy eating”, “fitness”, and “eating disorder recovery”. In addition, accounts linked to the discovered accounts were considered. Only public accounts posting in German language were contacted to request sharing of our study. Since most accounts did not agree to share the study, it was advertised on six Instagram accounts in the end.

During the initial screening, participants completed, in addition to the assessment of sociodemographic data, eating disorder diagnosis, and the DOS (described in the methods section), dietary preferences (i.e., vegan, vegetarian, omnivore, or other) and the following questionnaires: the Teruel Orthorexia Scale (TOS; Chace & Kluck, 2022), the Physical Activity Questionnaire (Fuchs et al., 2015), the Eating Disorder Examination-Questionnaire 8 (Kliem et al., 2016), the Rosenberg Self-Esteem Scale (Rosenberg, 2002), the WHO-5 Well-Being Index (Brähler et al., 2007), the Short Health Anxiety Inventory (Salkovskis et al., 2002), and the Multidimensional Body-Self Relations Questionnaire (Cash, 2018). Subsequently, participants reported about their last meal, current appetite and hunger, various food stimuli, and distress caused by the SARS-CoV-19. At the end of the screening, participants were invited to take part in two subsequent online experiments. To enable matching across study components, they were asked to generate a personal identification code.

The links to both online experiments were provided in the same email following the initial screening. Participants were instructed to complete the two sessions on different days and within one to two hours after a meal for standardization purposes. At the beginning of each experimental session, participants reported their current hunger, appetite, and details of their last meal. The first experimental session consisted of a line-orienting task and an affective priming task. The second experimental session started with questionnaires: the Food Cravings Questionnaire-Trait (Meule et al., 2014), the Salzburg Emotional Eating Scale (Meule et al., 2018a), the Salzburg Stress Eating Scale (Meule et al., 2018b), the short version of the General Depression Scale (Bailer et al., 2012), the restrained eating subscale from the Dutch Eating Behavior Questionnaire (Nagl et al., 2016; van Strien et al., 1986) and the Body Image Questionnaire-20 (Clement & Löwe, 1996). Then, participants completed the AAT and food image ratings. After that, they also rated additional food images and performed a food choice task and a food picture viewing task with desire to eat ratings after a negative emotion induction.

**References**

Bailer, M., Hautzinger, M., Hofmeister, D., & Keller, F. (2012). *Allgemeine Depressionsskala (ADS)*. https://digitalcollection.zhaw.ch/handle/11475/16613

Barthels, F., Meyer, F., & Pietrowsky, R. (2015). Die Düsseldorfer Orthorexie Skala–Konstruktion und Evaluation eines Fragebogens zur Erfassung ortho-rektischen Ernährungsverhaltens. *Zeitschrift für Klinische Psychologie und Psychotherapie*, *44*(2), 97–105. https://doi.org/10.1026/1616-3443/a000310

Brähler, E., Mühlan, H., Albani, C., & Schmidt, S. (2007). Teststatistische Prüfung und Normierung der deutschen Versionen des EUROHIS-QOL Lebensqualität-Index und des WHO-5 Wohlbefindens-Index. *Diagnostica*, *53*(2), 83–96. https://doi.org/10.1026/0012-1924.53.2.83

Cash, T. F. (2018). *Multidimensional Body-Self Relations Questionnaire* [Dataset]. https://doi.org/10.1037/t08755-000

Chace, S., & Kluck, A. S. (2022). Validation of the Teruel Orthorexia Scale and relationship to health anxiety in a U.S. sample. *Eating and Weight Disorders: EWD*, *27*(4), 1437–1447. https://doi.org/10.1007/s40519-021-01272-8

Clement, U., & Löwe, B. (1996). FKB-20—Fragebogen zum Körperbild. *hogrefe*. https://www.hogrefe.com/at/shop/fragebogen-zum-koerperbild.html

Fuchs, R., Klaperski, S., Gerber, M., & Seelig, H. (2015). Messung der Bewegungs- und Sportaktivität mit dem BSA-Fragebogen. *Zeitschrift für Gesundheitspsychologie*. (world). https://econtent.hogrefe.com/doi/10.1026/0943-8149/a000137

Kliem, S., Mößle, T., Zenger, M., Strauß, B., Brähler, E., & Hilbert, A. (2016). The eating disorder examination-questionnaire 8: A brief measure of eating disorder psychopathology (EDE-Q8). *The International Journal of Eating Disorders*, *49*(6), 613–616. https://doi.org/10.1002/eat.22487

Meule, A., Hermann, T., & Kübler, A. (2014). A short version of the Food Cravings Questionnaire—Trait: The FCQ-T-reduced. *Frontiers in Psychology*, *5*, 190. https://doi.org/10.3389/fpsyg.2014.00190

Meule, A., Reichenberger, J., & Blechert, J. (2018a). Development and Preliminary Validation of the Salzburg Emotional Eating Scale. *Frontiers in Psychology*, *9*. https://doi.org/10.3389/fpsyg.2018.00088

Meule, A., Reichenberger, J., & Blechert, J. (2018b). Development and preliminary validation of the Salzburg Stress Eating Scale. *Appetite*, *120*, 442–448. https://doi.org/10.1016/j.appet.2017.10.003

Nagl, M., Hilbert, A., Zwaan, M. de, Braehler, E., & Kersting, A. (2016). The German Version of the Dutch Eating Behavior Questionnaire: Psychometric Properties, Measurement Invariance, and Population-Based Norms. *PLOS ONE*, *11*(9), e0162510. https://doi.org/10.1371/journal.pone.0162510

Rosenberg, M. (2002). Self-Esteem Scale. *Zusammenstellung Sozialwissenschaftlicher Items Und Skalen (ZIS)*. https://doi.org/10.6102/zis46

Salkovskis, P. M., Rimes, K. A., Warwick, H. M. C., & Clark, D. M. (2002). The Health Anxiety Inventory: Development and validation of scales for the measurement of health anxiety and hypochondriasis. *Psychological Medicine*, *32*(5), 843–853. https://doi.org/10.1017/s0033291702005822

van Strien, T., Frijters, J. E. R., Bergers, G. P. A., & Defares, P. B. (1986). The Dutch Eating Behavior Questionnaire (DEBQ) for assessment of restrained, emotional, and external eating behavior. *International Journal of Eating Disorders*, *5*(2), 295–315. https://doi.org/10.1002/1098-108X(198602)5:2%253C295::AID-EAT2260050209%253E3.0.CO;2-T

**Appendix B: Sensitivity Analyses**

**Analyses with stronger Dissociation between AN, OEB, and CG**

To examine the robustness of the results to alternative group-assignment criteria, all primary analyses were repeated using stricter cut-offs. Because the DOS includes items that are inherent to both AN and orthorexia nervosa (e.g., setting of dietary rules), participants with elevated DOS scores could not be excluded from the AN group. However, stricter exclusion criteria were applied to the CG by additionally excluding individuals at risk for orthorexia nervosa (i.e., DOS ≥ 25 instead of ≥ 30; see Barthels et al., 2015). Furthermore, all participants with a past diagnosis of an eating disorder were excluded from the OEB group, dissociating AN and OEB as in the former group-assignment 12.5% of participants in OEB group had a past AN diagnosis. After applying these exclusions, the final sample comprised 92 CG, 29 OEB, and 35 AN participant. Generally, all findings mirror those in the main manuscript.

For liking ratings, all results are displayed in Table A1. As can be seen, differences between groups in liking ratings aligns with those in the main manuscript.

For approach bias, consistent with the main analyses, we found a significant 3-way interaction between the approach bias (Stimulus × Movement), and the presence of AN (vs. CG: *b* = -.06, *SE* = .03, *t*(150.50) = -2.30, *p* = .023), but no significant 3-way interaction between the approach bias, and the presence of OEB (vs. CG: *b* = -.01, *SE* = .03, *t*(150.98) = -0.47, *p* = .641) in the model including all food types. Further, separate models for each food type yielded results that also aligned with those reported in the main manuscript (see Table A2). Specifically, a significant approach bias difference between CG and AN was observed for high-calorie unhealthy foods at the Bonferroni-corrected alpha level, and for low-calorie healthy foods at the uncorrected alpha level.

Lastly, directly comparing AN and OEB (Stimulus × Movement × AN vs. OEB) did neither yield a significant difference in approach bias for all food types combined (*b* = -.05, *SE* = .03, *t*(61.94) = -1.47, *p* = .147), nor for the separate models for each food type (i.e., high-caloric healthy foods: *b* = -.02, *SE* = .04, *t*(61.07) = -0.66, *p* = .513; high-caloric unhealthy: *b* = -.07, *SE* = .04, *t*(62.00) = -1.67, *p* = .101; low-caloric healthy: *b* = -.06, *SE* = .04, *t*(62.16) = -1.47, *p* = .148). These findings align the main analysis.

**Table B1**

Differences between Groups in Explicit Liking Ratings

|  | **AN vs. CG** | | | | | **OEB vs. CG** | | | | **AN vs. OEB** | | | | |
| --- | --- | --- | --- | --- | --- | --- | --- | --- | --- | --- | --- | --- | --- | --- |
| *Food Category* | *b* | *SE* | *z* | *p* | *b* | | *SE* | *z* | *p* | | *b* | *SE* | *z* | *p* |
| All food stimuli | -.40 | .09 | -4.33 | **<.001** | -.15 | | .09 | -1.55 | .120 | | -.13 | .13 | **-0.98** | .328 |
| High-caloric unhealthy | -.74 | .16 | -4.71 | **<.001** | -.78 | | .17 | -4.62 | **<.001** | | -.04 | .24 | -.16 | .876 |
| High-caloric healthy | -.59 | .14 | -4.24 | **<.001** | 0 | | .15 | 0.01 | .988 | | -.60 | .22 | -2.72 | **.006** |
| Low-caloric healthy | .07 | .13 | **0.55** | .585 | .28 | | .14 | **2.02** | .044 | | -.20 | .16 | -1.26 | .207 |

*Note.* CG = control group, OEB = individuals with high levels of orthorexic eating behavior, AN = individuals with anorexia nervosa.

|  | **High-Caloric Healthy** | | | | | **High-Caloric Unhealthy** | | | |  | | **Low-Caloric Healthy** | | | |
| --- | --- | --- | --- | --- | --- | --- | --- | --- | --- | --- | --- | --- | --- | --- | --- |
| *Predictors* | *b* | *β* | *t* | *p* | *b* | | *β* | *t* | *p* | | *b* | | *β* | *t* | *p* |
| Movement | .07 | .19 | 7.23 | **<.001** | .07 | | .19 | 7.23 | **<.001** | | .07 | | .19 | 7.24 | **<.001** |
| Stimulus | -.05 | -.13 | -3.88 | **<.001** | -.04 | | -.10 | -2.82 | **.005** | | -.03 | | -.07 | -2.01 | **.044** |
| OEB | -.14 | -.37 | -3.04 | **.002** | -.14 | | -.37 | -3.07 | **.002** | | -.14 | | -.36 | -3.04 | **.002** |
| AN | -.17 | -.45 | -3.98 | **<.001** | -.17 | | -.45 | -3.98 | **<.001** | | -.17 | | -.44 | -3.98 | **<.001** |
| Movement × Stimulus | .12 | .32 | 7.66 | **<.001** | .12 | | .31 | 7.21 | **<.001** | | .13 | | .35 | 8.01 | **<.001** |
| Movement × OEB | -.02 | -.07 | -1.21 | .225 | -.02 | | -.06 | -1.17 | .242 | | -.02 | | -.06 | -1.19 | .232 |
| Movement × AN | .01 | .02 | 0.45 | .653 | .01 | | .02 | 0.44 | .660 | | .01 | | .02 | 0.46 | .648 |
| Stimulus × OEB | .03 | .08 | 1.62 | .106 | .01 | | .03 | 0.59 | .554 | | .00 | | .01 | 0.19 | .846 |
| Stimulus × AN | .03 | .08 | 1.89 | .059 | .05 | | .14 | 3.00 | **.003** | | .04 | | .10 | 2.05 | **.040** |
| Movement × Stimulus × OEB | -.01 | -.03 | -0.35 | .729 | -.02 | | -.04 | -0.49 | .622 | | -.01 | | -.02 | -0.26 | .797 |
| Movement × Stimulus × AN | -.04 | -.09 | -1.16 | .246 | -.08 | | -.22 | -2.75 | **.006** | | -.07 | | -.18 | -2.11 | **.035** |
| N | 156 _subject_  24 _Stimulus_ | | | | | 156 _subject_  24 _Stimulus_ | | | |  | | 156 _subject_ | | | |
|  |  |  |  |  |  |  |  |  |  |  | | 24 _Stimulus_ | | | |
| Observations | 27045 | | | | | 27010 | | | |  | | 27014 | | | |

**Table B2**

Interaction Between Approach Bias (Movement*Stimulus) and Participant Group per Food Type.

*Note.* AN = dummy variable for Control Group vs. individuals with anorexia nervosa, OEB = dummy variable Control Group vs. individuals with high levels of orthorexic eating behavior. *β* = standardized coefficient.

**Analyses dissociating groups based on BMI**

During revision, we identified that 12 CG and 5 OEB participants had a BMI below 18.5 (i.e., the underweight cut-off according to WHO and ICD-11), of which 4 CG and 1 OEB participants even had a BMI below 17 (i.e., cut-off for moderate AN according to the DSM-5). At the same time some ANs had a BMI above these thresholds, indicating partial overlap between groups, even though their group level BMI differed as expected based on diagnostic classification. As such overlap may potentially indicate misclassification (i.e., low-insight AN), we examined if it affected our findings by conducting two sensitivity analyses applying both BMI-based exclusion criteria: 1) we excluded participants with BMI ≥ 18.5 from the AN group and participants with BMI < 18.5 from the CG and OEB groups, and 2) excluding participants with BMI ≥ 17 from the AN group and BMI < 17 from the CG and OEB groups.

Findings for liking ratings are reported in Table A3 (BMI < 17 cut-off) and Table A4 (BMI < 18.5 cut-off). Findings for approach bias are reported in Table A5 (BMI < 17 cut-off) and Table A6 (BMI < 18.5 cut-off). Results remained consistent with those reported in the main manuscript which indicates that potential misclassification of a small number of participants did not affect the overall conclusions.

**Table B3**

Differences between Groups in Explicit Liking Ratings using a BMI cut-off of 17

|  | **AN vs. CG** | | | | | **OEB vs. CG** | | | | **AN vs. OEB** | | | | |
| --- | --- | --- | --- | --- | --- | --- | --- | --- | --- | --- | --- | --- | --- | --- |
| *Food Category* | *b* | *SE* | *z* | *p* | *b* | | *SE* | *z* | *p* | | *b* | *SE* | *z* | *p* |
| All food stimuli | -.45 | .09 | -4.50 | **<.001** | -.10 | | .08 | -1.30 | .192 | | -.24 | .11 | -2.16 | .031 |
| High-caloric unhealthy | -.83 | .17 | -5.02 | **<.001** | .62 | | .14 | -4.38 | **<.001** | | -.21 | .22 | -0.97 | .334 |
| High-caloric healthy | -.71 | .14 | -5.07 | **<.001** | 0 | | .12 | -.04 | .972 | | -.69 | .19 | -3.66 | **<.001** |
| Low-caloric healthy | .07 | .15 | 0.50 | .619 | .28 | | .13 | 2.19 | .029 | | -.19 | .16 | -1.24 | .216 |

*Note.* CG = control group, OEB = individuals with high levels of orthorexic eating behavior, AN = individuals with anorexia nervosa.

**Table B4**

Differences between Groups in Explicit Liking Ratings using a BMI cut-off of 18.5

|  | **AN vs. CG** | | | | | **OEB vs. CG** | | | | **AN vs. OEB** | | | | |
| --- | --- | --- | --- | --- | --- | --- | --- | --- | --- | --- | --- | --- | --- | --- |
| *Food Category* | *b* | *SE* | *z* | *p* | *b* | | *SE* | *z* | *p* | | *b* | *SE* | *z* | *p* |
| All food stimuli | -.45 | .09 | -4.85 | **<.001** | -.08 | | .09 | -0.93 | .355 | | -0.22 | 0.12 | -1.83 | .067 |
| High-caloric unhealthy | -.87 | .16 | -5.59 | **<.001** | -.58 | | .15 | -3.93 | **<.001** | | -0.28 | 0.22 | -1.31 | .192 |
| High-caloric healthy | -.66 | .15 | -4.53 | **<.001** | .03 | | .14 | 0.22 | .826 | | -0.69 | 0.22 | -3.2 | **.001** |
| Low-caloric healthy | 0.08 | .14 | 0.56 | .574 | .26 | | .14 | 1.91 | .057 | | -0.17 | 0.15 | -1.1 | .271 |

*Note.* CG = control group, OEB = individuals with high levels of orthorexic eating behavior, AN = individuals with anorexia nervosa.

**Table B5**

Interaction Between Approach Bias (Movement*Stimulus) and Group, BMI cut-off of 17

|  | **High-Caloric Healthy** | | | | | **High-Caloric Unhealthy** | | | |  | | **Low-Caloric Healthy** | | | |
| --- | --- | --- | --- | --- | --- | --- | --- | --- | --- | --- | --- | --- | --- | --- | --- |
| *Predictors* | *b* | *β* | *t* | *p* | *b* | | *β* | *t* | *p* | | *b* | | *β* | *t* | *p* |
| Movement | 0.08 | 0.20 | 7.81 | **<.001** | 0.08 | | 0.20 | 7.82 | **<.001** | | 0.08 | | 0.20 | 7.83 | **<.001** |
| Stimulus | -0.05 | -0.12 | -3.59 | **<.001** | -0.03 | | -0.09 | -2.51 | **.012** | | -0.02 | | -0.06 | -1.78 | .076 |
| OEB | -0.14 | -0.37 | -3.43 | **.001** | -0.14 | | -0.37 | -3.46 | **.001** | | -0.14 | | -0.37 | -3.44 | **.001** |
| AN | -0.17 | -0.45 | -3.61 | **<.001** | -0.17 | | -0.45 | -3.61 | **<.001** | | -0.17 | | -0.45 | -3.61 | **<.001** |
| Movement × Stimulus | 0.12 | 0.31 | 7.31 | **<.001** | 0.11 | | 0.29 | 6.69 | **<.001** | | 0.13 | | 0.35 | 8.16 | **<.001** |
| Movement × OEB | -0.02 | -0.06 | -1.14 | .255 | -0.02 | | -0.05 | -1.10 | .270 | | -0.02 | | -0.06 | -1.13 | .259 |
| Movement × AN | -0.01 | -0.02 | -0.30 | .761 | -0.01 | | -0.02 | -0.31 | .753 | | -0.01 | | -0.02 | -0.30 | .761 |
| Stimulus × OEB | 0.03 | 0.07 | 1.52 | .129 | 0.02 | | 0.04 | 0.85 | .397 | | 0.00 | | 0.01 | 0.18 | .854 |
| Stimulus × AN | 0.03 | 0.07 | 1.44 | .149 | 0.05 | | 0.13 | 2.34 | **.019** | | 0.03 | | 0.08 | 1.43 | .152 |
| Movement × Stimulus × OEB | -0.03 | -0.07 | -0.86 | .387 | -0.02 | | -0.05 | -0.66 | .511 | | -0.02 | | -0.04 | -0.53 | .595 |
| Movement × Stimulus × AN | -0.03 | -0.07 | -0.78 | .437 | -0.07 | | -0.19 | -2.04 | **.042** | | -0.05 | | -0.13 | -1.43 | .152 |
| N | 160 _subject_  24 _Stimulus_ | | | | | 160 _subject_  24 _Stimulus_ | | | |  | | 160 _subject_ | | | |
|  |  |  |  |  |  |  |  |  |  |  | | 24 _Stimulus_ | | | |
| Observations | 27718 | | | | | 27687 | | | |  | | 27689 | | | |

*Note.* AN = dummy variable for Control Group vs. individuals with anorexia nervosa, OEB = dummy variable Control Group vs. individuals with high levels of orthorexic eating behavior. *β* = standardized coefficient.

**Table B6**

Interaction Between Approach Bias (Movement*Stimulus) and Group, BMI cut-off of 18.5

|  | **High-Caloric Healthy** | | | | | **High-Caloric Unhealthy** | | | |  | | **Low-Caloric Healthy** | | | |
| --- | --- | --- | --- | --- | --- | --- | --- | --- | --- | --- | --- | --- | --- | --- | --- |
| *Predictors* | *b* | *β* | *t* | *p* | *b* | | *β* | *t* | *p* | | *b* | | *β* | *t* | *p* |
| Movement | .08 | .21 | 7.52 | **<.001** | .08 | | .21 | 7.53 | **<.001** | | .08 | | .21 | 7.53 | **<.001** |
| Stimulus | -.05 | -.12 | -3.56 | **<.001** | -.03 | | -.09 | -2.38 | **.017** | | -.02 | | -.06 | -1.61 | .108 |
| OEB | -.16 | -.43 | -3.80 | **<.001** | -.16 | | -.43 | -3.82 | **<.001** | | -.16 | | -.43 | -3.80 | **<.001** |
| AN | -.16 | -.42 | -3.57 | **<.001** | -.16 | | -.42 | -3.57 | **<.001** | | -.16 | | -.42 | -3.57 | **<.001** |
| Movement × Stimulus | .12 | .32 | 7.21 | **<.001** | .11 | | .29 | 6.39 | **<.001** | | .13 | | .34 | 7.57 | **<.001** |
| Movement × OEB | -.03 | -.07 | -1.28 | .201 | -.02 | | -.07 | -1.25 | .210 | | -.03 | | -.07 | -1.27 | .203 |
| Movement × AN | 0 | .01 | 0.19 | .853 | 0 | | .01 | 0.18 | .860 | | 0 | | .01 | 0.19 | .847 |
| Stimulus × OEB | .02 | .06 | 1.32 | .188 | .01 | | .02 | 0.50 | .618 | | 0 | | 0 | -0.08 | .939 |
| Stimulus × AN | .04 | .11 | 2.21 | **.027** | .05 | | .14 | 2.66 | **.008** | | .04 | | .10 | 1.82 | .069 |
| Movement × Stimulus × OEB | -.02 | -.05 | -0.55 | .580 | 0 | | -.01 | -0.07 | .943 | | 0 | | -.01 | -0.14 | .890 |
| Movement × Stimulus × AN | -.04 | -.11 | -1.27 | .204 | -.08 | | -.21 | -2.30 | **.021** | | -.06 | | -.16 | -1.75 | .079 |
| N | 153 _subject_  24 _Stimulus_ | | | | | 153 _subject_  24 _Stimulus_ | | | |  | | 153 _subject_ | | | |
|  |  |  |  |  |  |  |  |  |  |  | | 24 _Stimulus_ | | | |
| Observations | 26577 | | | | | 26570 | | | |  | | 26547 | | | |

*Note.* AN = dummy variable for Control Group vs. individuals with anorexia nervosa, OEB = dummy variable Control Group vs. individuals with high levels of orthorexic eating behavior. *β* = standardized coefficient.

**Analyses including age as a covariate**

As the three groups differed significantly in age, next to expected differences in BMI and DOS, we included age as a covariate to control for its potential influence on our findings. Specifically, for the liking models, age was included as a fixed effect as follows:

| (1) | *LikingRatings ~ Dummy 1 (CG vs. AN) + Dummy 2 (CG vs. OEB) +* ***age*** *+ (1 \| Subject) + (1 \| Stimulus)* |
| --- | --- |

For the approach bias analyses, interactions between movement, stimulus, and age were included. This allowed us to account not only for age-related differences in overall reaction times but also for potential age-related differences in approach bias:

| (2) | *inv.RTs ~ Movement (avoid vs. approach)* × *Stimulus (object vs. food)* × *Dummy 1 (CG vs. AN) + Movement (avoid vs. approach)* × *Stimulus (object vs. food)* × *Dummy 2 (CG vs. OEB) +* ***Movement (avoid vs. approach)* × *Stimulus (object vs. food)* × *age***  *(Movement* × *Stimulus* × *Group \| Subject) +*  *(1 \| Stimulus)* |
| --- | --- |

As can be seen in Table A7, findings for liking ratings are robust against the inclusion of age as a covariate.

**Table B7**

Differences between Groups in Explicit Liking Ratings when Controlling for Age.

|  | **AN vs. CG** | | | | | **OEB vs. CG** | | | | **AN vs. OEB** | | | | |
| --- | --- | --- | --- | --- | --- | --- | --- | --- | --- | --- | --- | --- | --- | --- |
| *Food Category* | *b* | *SE* | *z* | *p* | *b* | | *SE* | *z* | *p* | | *b* | *SE* | *z* | *p* |
| All food stimuli | -.36 | .09 | -3.83 | **<.001** | -.08 | | .09 | -0.86 | .388 | | -.15 | .11 | -1.30 | .192 |
| High-caloric unhealthy | -.70 | .16 | -4.43 | **<.001** | -.56 | | .16 | -3.57 | **<.001** | | -.07 | .22 | -0.33 | .742 |
| High-caloric healthy | -.55 | .14 | -3.83 | **<.001** | -.02 | | .14 | -0.16 | .877 | | -.49 | .21 | -2.36 | **.018** |
| Low-caloric healthy | .20 | .13 | 0.72 | 0.470 | .28 | | .13 | 2.15 | .031 | | -.19 | .15 | -1.23 | .219 |

*Note.* CG = control group, OEB = individuals with high levels of orthorexic eating behavior, AN = individuals with anorexia nervosa.

**Table B8**

Differences between Groups in Approach Bias when Controlling for Age.

|  | **High-Caloric Healthy** | | | | | **High-Caloric Unhealthy** | | | |  | | **Low-Caloric Healthy** | | | |
| --- | --- | --- | --- | --- | --- | --- | --- | --- | --- | --- | --- | --- | --- | --- | --- |
| *Predictors* | *b* | *β* | *t* | *p* | *b* | | *β* | *t* | *p* | | *b* | | *β* | *t* | *p* |
| Movement | .12 | .20 | 5.00 | **<.001** | .12 | | .20 | 5.00 | **<.001** | | .12 | | .20 | 5.02 | **<.001** |
| Stimulus | -.05 | -.12 | -2.36 | **.018** | -.05 | | -.09 | -2.09 | **.037** | | -.03 | | -.06 | -1.12 | .264 |
| OEB | -.08 | -.22 | -2.15 | **.031** | -.08 | | -.22 | -2.18 | **.029** | | -.08 | | -.22 | -2.16 | **.031** |
| AN | -.17 | -.46 | -4.54 | **<.001** | -.17 | | -.46 | -4.54 | **<.001** | | -.17 | | -.46 | -4.54 | **<.001** |
| Age | -.01 | -.22 | -5.44 | **<.001** | -.01 | | -.22 | -5.44 | **<.001** | | -.01 | | -.22 | -5.44 | **<.001** |
| Movement × Stimulus | .14 | .31 | 3.74 | **<.001** | .15 | | .29 | 3.92 | **<.001** | | .14 | | .34 | 3.65 | **<.001** |
| Movement × OEB | -.01 | -.03 | -0.69 | .492 | -.01 | | -.03 | -0.65 | .513 | | -.01 | | -.03 | -0.68 | .496 |
| Movement × AN | 0 | 0 | 0.10 | .921 | 0 | | 0 | 0.09 | .930 | | 0 | | .01 | 0.10 | .917 |
| Stimulus × OEB | .02 | .06 | 1.41 | .160 | .02 | | .04 | 0.85 | .396 | | 0 | | -.01 | -0.12 | .908 |
| Stimulus × AN | .03 | .08 | 1.78 | .075 | .05 | | .14 | 2.89 | **.004** | | .03 | | .09 | 1.85 | .065 |
| Movement × Age | 0 | -.04 | -1.87 | .062 | 0 | | -.04 | -1.86 | .063 | | 0 | | -.04 | -1.87 | .061 |
| Stimulus × Age | 0 | .01 | 0.37 | .711 | 0 | | .01 | 0.73 | .466 | | 0 | | 0 | 0.22 | .829 |
| Movement × Stimulus × OEB | -.02 | -.06 | -0.69 | .491 | -.02 | | -.04 | -0.51 | .612 | | -.01 | | -.02 | -0.22 | .827 |
| Movement × Stimulus × AN | -.03 | -.08 | -1.04 | .299 | -.08 | | -.21 | -2.58 | **.010** | | -.06 | | -.16 | -1.98 | **.048** |
| Movement × Stimulus × Age | 0 | -.02 | -0.72 | .474 | 0 | | -.04 | -1.16 | .246 | | 0 | | -.01 | -0.37 | .711 |
| N | 175 _subject_  24 _Stimulus_ | | | | | 175 _subject_  24 _Stimulus_ | | | |  | | 175 _subject_ | | | |
|  |  |  |  |  |  |  |  |  |  |  | | 24 _Stimulus_ | | | |
| Observations | 30337 | | | | | 30314 | | | |  | | 30307 | | | |

*Note.* AN = dummy variable for Control Group vs. individuals with anorexia nervosa, OEB = dummy variable Control Group vs. individuals with high levels of orthorexic eating behavior. *β* = standardized coefficient.

**Appendix C**

**List of all Food Stimuli included in the respective Categories**

Category 1: low-caloric and healthy foods, namely oranges (34 kcal), salad (31 kcal), strawberries (31 kcal), tomatoes (16 kcal), blueberries (43 kcal), and cucumber (12 kcal)

Category 2**:** high-caloric and unhealthy foods, namely fries (165 kcal), pizza (240 kcal), cake (179 kcal), milk chocolate (535 kcal), ice-cream (211 kcal), peanut crisps (500 kcal)

Category 3: high-caloric and healthy foods, namely avocado (217 kcal), dates (285 kcal), nuts (662 kcal), vegetable sandwich (204 kcal), granola bar (411 kcal) and wrap (172 kcal)

**Appendix D**

**Calorie and Healthiness Ratings separately for CG, AN, and OEB**

In two seperate models, healthiness and calorie ratings were predicted with an interaction between stimulus type (HH = high-caloric healthy foods, HU = high-caloric unhealthy foods, LH = low-caloric healthy foods) and group status (CG = control group, OEB = individuals with high levels of orthorexic eating behavior, AN = individuals with anorexia nervosa) using a multilevel model with random intercepts per stimulus and participant. In Figure D1, you see the estimated marginal means of calorie and healthiness ratings for each stimulus and group. The results for pairwise contrasts are shown in Table D1 to Table D4. Importantly, HH and LH were rated as healthier than HU across all three groups (Table D1), and there were no significant differences in healthiness ratings for the healthy food types (i.e., HH and LH) between groups besides that AN rated HU as less healthy than the CG (Table D3).

**Figure D1**

Differences in calorie and healthiness ratings between the three participant groups

*Note.* HH = high-caloric healthy foods, HU = high-caloric unhealthy foods, LH = low-caloric healthy foods, CG = control group, OEB = individuals with high levels of orthorexic eating behavior, AN = individuals with anorexia nervosa. The error bars represent confidence intervals.

**Table D1**

Differences between Stimuli in Explicit Healthiness Ratings for each group

|  | **HH vs. HU** | | | | **HH vs. LH** | | | | **HU vs. LH** | | | |
| --- | --- | --- | --- | --- | --- | --- | --- | --- | --- | --- | --- | --- |
|  | *Odds Ratio* | *SE* | *z* | *p* | *Odds Ratio* | *SE* | *z* | *p* | *Odds Ratio* | *SE* | *z* | *p* |
| CG | 12.28 | 3.15 | 9.77 | **< .001** | 0.25 | 0.06 | -5.46 | **< .001** | 0.02 | 0.01 | -15.15 | **< .001** |
| OEB | 12.70 | 3.35 | 9.64 | **< .001** | 0.20 | 0.05 | -6.05 | **< .001** | 0.02 | 0.00 | -15.53 | **< .001** |
| AN | 16.76 | 4.45 | 10.62 | **< .001** | 0.21 | 0.06 | -5.87 | **< .001** | 0.01 | 0.00 | -16.28 | **< .001** |

*Note.* HH = high-caloric healthy foods, HU = high-caloric unhealthy foods, LH = low-caloric healthy foods, CG = control group, OEB = individuals with high levels of orthorexic eating behavior, AN = individuals with anorexia nervosa.

**Table D2**

Differences between Stimuli in Explicit Calorie Ratings for each group

|  | **HH vs. HU** | | | | **HH vs. LH** | | | | **HU vs. LH** | | | |
| --- | --- | --- | --- | --- | --- | --- | --- | --- | --- | --- | --- | --- |
|  | *Odds Ratio* | *SE* | *z* | *p* | *Odds Ratio* | *SE* | *z* | *p* | *Odds Ratio* | *SE* | *z* | *p* |
| CG | 0.28 | 0.06 | -5.86 | **< .001** | 9.48 | 2.05 | 10.40 | **< .001** | 33.69 | 7.34 | 16.16 | **< .001** |
| OEB | 0.38 | 0.09 | -4.28 | **.001** | 14.60 | 3.27 | 11.97 | **< .001** | 38.10 | 8.63 | 16.08 | **< .001** |
| AN | 0.48 | 0.11 | -3.27 | **.030** | 23.82 | 5.39 | 14.00 | **< .001** | 49.98 | 11.43 | 17.11 | **< .001** |

*Note.* HH = high-caloric healthy foods, HU = high-caloric unhealthy foods, LH = low-caloric healthy foods, CG = control group, OEB = individuals with high levels of orthorexic eating behavior, AN = individuals with anorexia nervosa.

**Table D3**

Differences between Groups in Explicit Healthiness Ratings

|  | **AN vs. CG** | | | | **OEB vs. CG** | | | | **AN vs. OEB** | | | |
| --- | --- | --- | --- | --- | --- | --- | --- | --- | --- | --- | --- | --- |
| *Food Category* | *Odds Ratio* | *SE* | *z* | *p* | *Odds Ratio* | *SE* | *z* | *p* | *Odds Ratio* | *SE* | *z* | *p* |
| High-caloric unhealthy | 1.52 | 0.15 | 4.22 | **0.001** | 1.22 | 0.12 | 2.08 | 0.486 | 1.25 | 0.15 | 1.90 | 0.616 |
| High-caloric healthy | 1.12 | 0.10 | 1.21 | 0.956 | 1.18 | 0.10 | 1.86 | 0.639 | 0.95 | 0.10 | -0.50 | 1.000 |
| Low-caloric healthy | 0.96 | 0.10 | -0.43 | 1.000 | 0.97 | 0.09 | -0.32 | 1.000 | 0.99 | 0.12 | -0.10 | 1.000 |

*Note.* CG = control group, OEB = individuals with high levels of orthorexic eating behavior, AN = individuals with anorexia nervosa.

**Table D4**

Differences between Groups in Explicit Calorie Ratings

|  | **AN vs. CG** | | | | **OEB vs. CG** | | | | **AN vs. OEB** | | | |
| --- | --- | --- | --- | --- | --- | --- | --- | --- | --- | --- | --- | --- |
| *Food Category* | *Odds Ratio* | *SE* | *z* | *p* | *Odds Ratio* | *SE* | *z* | *p* | *Odds Ratio* | *SE* | *z* | *p* |
| High-caloric unhealthy | 0.83 | 0.08 | -1.93 | 0.589 | 0.95 | 0.09 | -0.55 | 1.000 | 0.87 | 0.10 | -1.19 | 0.958 |
| High-caloric healthy | 0.49 | 0.05 | -7.75 | **<.001** | 0.70 | 0.06 | -4.13 | **0.001** | 0.70 | 0.08 | -3.27 | **0.030** |
| Low-caloric healthy | 1.22 | 0.12 | 2.10 | 0.476 | 1.07 | 0.10 | 0.77 | 0.998 | 1.14 | 0.13 | 1.15 | 0.966 |

*Note.* CG = control group, OEB = individuals with high levels of orthorexic eating behavior, AN = individuals with anorexia nervosa.

**Appendix E**

**Within-subject correlations between image ratings per diagnosis group**

Exploratory multilevel correlations (Figure 3) *on the image level* examined whether within individuals, specific perceptions of calorie content and healthiness of a given food (i.e., foods rated higher or lower than an that individual’s average) was related to food-specific liking of that food. Results showed that both AN and OEB liked foods perceived as higher in calories less (r > -.30). In contrast, the CG showed only a small negative association (r > .10). Additionally, in OEB, perceived healthiness was strongly and positively related to liking (r > .50), a link that was weaker in AN (r > .30) and CG (r > .10). Together, these findings may indicate that AN and OEB generally dislike high-caloric foods more than CG, but that this pattern does not extend to high-calorie healthy foods in OEB because the positive influence of perceived healthiness appeared to offset the negative effect of higher calorie content.

**Figure E1**

Multilevel Within-participant Correlations between Food-specific Liking Ratings and Perceived Healthiness and Calorie Content

*Note.* CG = control group, OEB = individuals with high levels of orthorexic eating behavior, AN = individuals with anorexia nervosa.
